# Blood transfusion in the critically ill: does storage age matter?

**DOI:** 10.1186/1757-7241-17-35

**Published:** 2009-08-13

**Authors:** Marianne J Vandromme, Gerald McGwin, Jordan A Weinberg

**Affiliations:** 1 Department of Surgery, Center for Injury Sciences, University of Alabama at Birmingham, Birmingham, AL, USA

## Abstract

Morphologic and biochemical changes occur during red cell storage prior to product expiry, and these changes may hinder erythrocyte viability and function following transfusion. Despite a relatively large body of literature detailing the metabolic and structural deterioration that occurs during red cell storage, evidence for a significant detrimental clinical effect related to the transfusion of older blood is relatively less conclusive, limited primarily to observations in retrospective studies. Nonetheless, the implication that the transfusion of old, but not outdated blood may have negative clinical consequences demands attention. In this report, the current understanding of the biochemical and structural changes that occur during storage, known collectively as the storage lesion, is described, and the clinical evidence concerning the detrimental consequences associated with the transfusion of relatively older red cells is critically reviewed. Although the growing body of literature demonstrating the deleterious effects of relatively old blood is compelling, it is notable that all of these reports have been retrospective, and most of these studies have evaluated patients who received a mixture of red cell units of varying storage age. Until prospective studies have been completed and produce confirmative results, it would be premature to recommend any modification of current transfusion practice regarding storage age.

In 1917, Frances Payton Rous and J.R. Turner identified that a citrate-glucose solution allowed for the preservation of a whole blood unit for up to five days, thus facilitating the formative practice of blood banking[1]. Later, Loutit and Mollison of Great Britain developed the first anticoagulant of the modern era, known as acid-citrate-dextrose (ACD)[1]. ACD extended the shelf life of refrigerated blood to 21 days, and ACD remained in wide spread usage until the 1960s, when it was replaced by citrate-phosphate-dextrose (CPD) and citrate-phosphate-dextrose-adenine (CPDA) solutions that increased shelf life to 35 days and 42 days respectively. More recently, additive solutions containing saline, adenine, and dextrose have been developed to augment red cell survival following transfusion, although without any direct increase in storage duration[1,2].

It is now well appreciated, however, that a number of morphologic and biochemical changes occur during red cell storage prior to product expiry, and these changes may hinder erythrocyte viability and function following transfusion. Despite a relatively large body of literature detailing the metabolic and structural deterioration that occurs during red cell storage, evidence for a significant detrimental clinical effect related to the transfusion of older blood is relatively less conclusive, limited primarily to observations in retrospective studies. Nonetheless, the implication that the transfusion of old, but not outdated blood may have negative clinical consequences demands attention. The purpose of this report is to describe the current understanding of the biochemical and structural changes that occur during storage, known collectively as the storage lesion, and to critically review the clinical evidence concerning the detrimental consequences associated with the transfusion of relatively older red cells.

## The Storage Lesion

The term "storage lesion" has been traditionally used to describe the progressive degradation of red cell structure and function that occurs during conventional red cell storage. It is useful, however, to also consider the accumulation of bioreactive substances that occur during storage under the umbrella of the storage lesion, as these substances may not be innocuous when transfused (Table 1). Although the components of the storage lesion are well described, the clinical relevance of these storage related changes remains uncertain.

**Table 1:** Characteristics of the storage lesion.

**Storage Effect**	**Consequences**
*Changes to red cell structure and function*	

**Cellular membrane changes**	Erythrocytes change shape Decreased survivability
**Decreased 2,3-diphsophoglycerate**	Increased oxygen affinity Decreased oxygen delivery
**Decreased adenosine triphosphate**	Erythrocytes change shape Increased cell fragility Less resistance to oxidative stress
*Changes in red cell storage medium*	
**Accumulation of bioactive substances** **(cytokines, histamines, lipids, enzymes)**	Increased oxidative environment Febrile transfusion reactions Immunologic activation/suppression

### Changes to red cell structure and function

After 14 days of storage, byproducts of glycolytic metabolism, lactic acid, and proteins accumulate. These byproducts, which in vivo are readily removed from circulation, linger and result in structural and functional changes. As storage time extends past 14 days, the red cells become less pliable and therefore unable to traverse small vessels of the microcirculation, ultimately resulting in decreased oxygen delivery because the oxygenated red cells cannot traverse the end-organ capillary beds. The change in shape from standard biconcave disks to spiculated echinocytic erythrocytes also makes the cells more aggregable, increasing the likelihood of occluding the microcirculation, leading to tissue ischemia[3]. It is notable, however, that these observations pertain to research performed with whole blood, rather than red cell concentrates. In leukodepleted red cell concentrates, Raat et al. found no significant red cell deformation following six weeks of storage[4]. The mechanism associated with the membrane changes leading to inability to maintain structure and stiffening is likely related to the failure to maintain the cytoskeleton, which is independent of adenosine triphosphate (ATP) levels, since cellular membrane changes are observed prior to the cellular decrease in ATP[3,5].

While structural changes are observed on the red cell surface as storage time increases, biochemical changes occur intracellularly, with decreases in enzymes and stored energy concentrations that affect red blood cell function. The metabolite and enzymatic regulator of hemoglobin, 2,3-diphsophoglycerate (2,3 DPG), has been shown to decrease to near non-detectible levels within two weeks of storage[6]. The decreased concentration in 2,3-DPG leads to significant increases in hemoglobin's affinity for oxygen, which ultimately decreases oxygen delivery to the peripheral tissues upon re-infusion, because oxygen will not unbind from hemoglobin. The red cell devoid of 2,3-DPG can recover its normal levels within 72 hours after infusion, and no irreversible effect in the function of the red cell has been observed[7]. Given the delay to complete recovery of ideal enzymatic function and oxygen unloading in the peripheral tissue, the desired augmentation of oxygen delivery following transfusion is not immediate, but rather potentially delayed until 2,3-DPG levels are normalized intracellularly[3].

ATP is not only an intracellular source of energy, but has more recently been associated with vasodilation in hypoxic conditions, whereby ATP is released from the RBC and ultimately initiates a signaling cascade that stimulates nitric oxide production[6]. As ATP levels decrease during storage, active transport, antioxidant reactions, and membrane phospholipid distribution and other energy requiring reactions decrease, causing the cell to become more vulnerable to a stressing environment. Recent studies have shown as much as a 60% decrease in intracellular ATP levels with storage greater than 5 weeks[6]. In a study by Raat et al., increasing intracellular ATP levels improved oxygen capacity, suggesting that ATP concentration (and indirectly storage age) affects cellular oxygen carrying capacity and oxygen delivery[4].

### Changes in red cell storage medium

The first evidence of an immunomodulative effect associated with allogeneic blood transfusion was documented by Opelz et al. in 1973, when improved graft function and a lower incidence of graft rejection were observed in renal transplant recipients who received a blood transfusion prior to transplantation[8]. Immunomodulation related to blood transfusion is presumed to be related to the accumulation of various soluble bioactive substances, primarily but not exclusively derived from passenger leukocytes contained in the donor unit. This effect can vary from immunosupression, as evident by increased association with nosocomial and post-operative infections, increased cancer recurrence, and enhanced allograft survival, to immune activation, as evident by hemolytic transfusion reactions, transfusion-associated graft-versus-host disease, transfusion-related lung injury, and possible autoimmune diseases[9]. Although the mechanism of immunomodulation is largely unknown, the infusion of the degradative components of passenger leukocytes, including histamine, lipids, cytokines, and human leukocyte antigen (HLA) is implicated. The bioactive soluble molecules are released from the leukocytes during storage and accumulate as storage time lengthens[10].

As the passenger leukocytes contained in a typical red cell unit are implicated in the deleterious effect of older blood, it follows that leukoreduction of red cell concentrates (which is performed on a universal basis in many countries), might mitigate this effect. Biffl et al., however, observed that although the plasma from nonleukoreduced aged stored blood delayed neutrophil apoptosis (a proinflammatory phenomenon) and primed neutrophils for cytotoxicity, plasma from stored blood that had undergone prestorage leukoreduction did not, in fact, modify this effect[11]. Immunization is proposed to be related to HLA-DR compatibility and when at least one antigen from the donor matches the recipient, immune tolerance is observed; on the contrary, when there is complete mismatch of HLA-DR antigens, immunization is activated[12].

### Effect of storage on tissue oxygenation

In clinical practice, blood is often transfused in an effort to augment tissue oxygen delivery. Nonetheless, there is some question as to the effectiveness of transfusion in this regard, particularly related to red cell storage. Raat et al. demonstrated that blood stored for longer periods of time (5 weeks) resulted in diminished oxygen delivery capacity to the gut microcirculation of anemic oxygen-supply dependent rats, while relatively fresh blood, stored only several days, and intermediate-aged blood, stored several weeks, improved oxygen delivery[4]. Likewise, Fitzgerald et al. demonstrated that blood stored for 28 days did not improve oxygen delivery and consumption when transfused to rats while transfusion of blood stored only 3 days did, in fact, improve oxygen delivery[13]. These observations in rats, however, may not be extrapolative to humans. Rat red cells age approximately four times faster than human red cells in storage, and fail to regenerate 2,3-DPG when treated with a rejuvenation solution, in contrast to human red cells[14].

In human studies, observations have been less conclusive. Marik et al. observed a decreased gastric pH, a measure of gastric mucosal oxygenation status, in patients receiving blood that had been stored beyond 15 days[15]. Walsh et al., however, were unable to replicate the results of Marik et al, which were identified in the course of *post hoc *analysis. In a prospective, double-blind trial of critically ill intensive care unit patients randomized to receive leukodepleted red cells stored either ≤ 5 days or ≥ 20 days, Walsh et al. observed no significant differences in gastric pH measurements or other indices of global tissue oxygenation[16]. Recently, Kiraly et al. evaluated peripheral tissue oxygenation as measured by near infrared spectroscopy during the course of red cell transfusion[17]. The authors observed that patients transfused with blood stored 21 days or longer had a statistically significant decline in tissue oxygen saturation compared with those transfused with blood less than 21 days old. Whether or not the magnitude of the observed decline is clinically meaningful in any way remains uncertain.

## Red Cell Storage and Clinical Outcomes

The association between the transfusion of relatively older blood and morbidity and mortality has been demonstrated in multiple retrospective studies utilizing various study designs (Table 2). In 1997, Purdy et al. reported the association between blood storage age and survival among 31 septic ICU patients[18]. No differences were observed between survivors and nonsurvivors concerning age, gender, ICU length of stay, APACHE II score, or total number of red cell units transfused. However, the median age of red cell units transfused to survivors during sepsis was 17 days (range 5-35) versus 25 days (range 9-36) for nonsurvivors (P < 0.0001).

**Table 2:** Clinical outcome studies reviewing the effects of red cell storage age, in order of publication

**Author**	**Study Population**	**No. Patients**	**Major Conclusion**
**Purdy et al.**[18]	Septic ICU patients	31	Patients who died received older RBC
**Vamvakas et al.**[31]	CABG patients	416	Transfusion of RBC with longer storage time associated with pneumonia
**Zallen et al.**[19]	Trauma patients who received 6-20 RBC in the first 12 hours post-injury	63	Patients who developed MOF received older blood (30 vs. 24 days)
**Vamvakas et al.**[32]	CABG patients	268	Transfusion of old RBC was not associated with increased morbidity or mortality
**Offner et al.**[20]	Trauma patients who received 6-20 RBC in the first 12 hours post-injury	62	Transfusion of old blood was associated with increase risk of infection
**Keller et al.**[21]	Trauma patients who received ≥1 RBC within 48 hours of admission	86	Older RBC were associated with longer hospital length of stay
**Murrell et al.**[33]	Trauma patients who received ≥1 RBC	275	Patients who received older RBC had longer length of ICU stay but no increased in-hospital mortality
**Koch et al.**[24]	CABG patients who received exclusively young or old blood	6,002	Patients receiving old RBC had higher mortality (short and long term)
**Weinberg et al.**[22]	Trauma patients who received ≥1 RBC within the first 24 hours post-injury	1,813	Blood storage age potentiated the increased odd of mortality seen with larger volumes of transfusion
**Weinberg et al. **[23]	Less severely injured trauma patients who received no RBC in the first 48 hours post-injury	1,624	Transfusion of old blood was associated with increased mortality, renal failure, and pneumonia

RBC = red blood cell unit

In 1999, Zallen et al. examined the association between red cell storage age and multiple organ failure (MOF) in a matched case-control study concerning trauma patients that received between 6 and 20 units of red cells in the first 12 hours following injury[19]. No difference in ISS or transfusion requirement was observed between MOF positive (n = 23) and MOF negative (n = 40) groups. The authors identified that the mean age of transfused blood was significantly greater in the MOF positive patients (30.5+/-1.6 days versus 24+/-0.5 days). Multivariate analysis identified mean age of blood, number of units older than 14 days, and number of units older than 21 days as independent risk factors for MOF.

From the same institution, Offner et al. evaluated the association between transfusion of relatively older blood and post-injury infection in a similar patient cohort[20]. In this study of 61 patients who each received between 6 and 20 units of red cells in the first 12 hours after injury, patients who developed infections were found to have received 11.7 +/- 1.0 and 9.9 +/- 1.0 units of red cells older than 14 and 21 days, respectively, compared with 8.7 +/- 0.8 and 6.7 +/- 0.08 units in patients who did not develop infections (both p < 0.05). Multivariate analysis demonstrated age of blood to be an independent risk factor for post-injury infection.

Keller et al. examined the influence of blood storage age on hospital length of stay in a trauma patient cohort of 86 patients[21]. Univariate analysis demonstrated that the number of units of transfused blood older than 14 days correlated significantly with hospital length of stay. They also evaluated the total number of units transfused, mean age of blood, age of the oldest unit, and average of the two oldest units for associations with length of stay; none correlated significantly with length of stay. Multivariate analysis was then performed and indicated that the number of units of blood older than 14 days remained significantly associated with increased length of stay.

As evident in the studies described above, the evaluation of the independent role of storage age on outcomes in patient populations that received a heterogeneous distribution of relatively old and young blood is far from straightforward. Utilization of measures of central tendency, such as the mean or median age of all units transfused to a given patient, may simplify the analysis from a statistical standpoint, but is flawed in that it makes an assumption of mechanism, whereby the transfusion of relatively younger units engenders a protective (or a watering down) effect, offsetting the proposed deleterious effect of older blood. Given the present understanding of the storage lesion, there is no evident rationale for the assumption of such. Alternatively, analyses that focus on the volume of old blood transfused, while avoiding this assumption of mechanism, are hindered by the confounding of total transfusion volume. The observed associations between the transfusion of relatively older blood and morbidity or mortality may actually be more reflective of the residual effect of total transfusion volume rather than blood storage age, as transfusion volume and transfusion storage age are necessarily linked variables. The more units of blood a patient receives the greater likelihood that the mean age of those units will be older. Further, given that receipt of larger units of blood likely reflects more serious injuries, and therefore a greater likelihood of morbidity and mortality, any adverse association with older mean age may simply reflect higher injury severity. It is therefore important to consider not only the age of the blood transfused but also the volume and these measures should not be treated in an independent manner. If the associations between older blood and outcomes as outlined above were actually secondary to the residual confounding of transfusion volume, the associations between outcome and the volume of young blood transfused would be expected to be similar.

With this in mind, we recently evaluated the association between mortality and the transfusion of both older and younger blood, respectively[22]. Among 1,813 severely injured patients (mean ISS 26) admitted to the trauma service of the University of Alabama at Birmingham University Hospital, who received one or more units of blood within the initial 24 hours of hospitalization, we determined that while larger volumes of blood, irrespective of storage age, were associated with an increased odds of mortality, the transfusion of blood stored beyond 14 days appeared to significantly potentiate this association, suggesting the existence of a veritable association between storage age and outcome.

In a second study, we evaluated the relationship between blood storage age and adverse outcomes in a relatively less injured population[23]. This cohort of 1,624 trauma patients comprised those with blunt mechanism of injury, ISS < 25, and no blood transfusions administered within the first 48 hours of hospital admission. Similar to our previous work, we determined the effect of both young and old blood on outcome, respectively. We observed that the receipt of old blood was significantly associated with mortality, acute renal dysfunction, and pneumonia, whereas the receipt of young blood was not, further suggesting that transfusion of older blood is independently associated with outcome, even in relatively less severely injured patients.

Nonetheless, the methodological difficulties presented by patients receiving a heterogeneous distribution of old and young blood remain; analyses limited to those patients that received exclusively old versus exclusively young blood may simplify things considerably. In our study concerning 1,813 trauma patients, we performed a subgroup analysis concerning only those patients who received exclusively young or old blood, and found that among those patients receiving a total of 3 or more red cell units, receipt of old blood was associated with an over 2-fold increased odds of death[22]. Koch et al. performed a similar analysis concerning 6,002 cardiac surgery patients, and observed that patients in the older blood group had significantly higher incidences of in-hospital mortality, intubation beyond 72 hours, renal failure, and sepsis[24]. Again, however, the coupling of storage age and volume of transfusion must be acknowledged. Although the distribution of transfusion volume in both the young and old groups in this study (and in our subgroup analysis) as represented by the mean was similar between groups, it remains plausible that transfusion volume remains a relevant residual confounder. In fact, the report by Koch et al. has been vocally criticized for failure to adequately account for multiple potential confounders including differences concerning total transfusion volume, underlying comorbidities, and ABO blood groups between groups[25,26].

It is notable that in our reported experience described above, all patients were transfused with blood that had undergone prestorage leukoreduction[22,23]. Although leukoreduction has well documented efficacy related to specific clinical circumstances, a generalized benefit remains unproven[27]. Indeed, Nathens et al. performed a randomized trial comparing prestorage leukoreduced versus standard nonleukoreduced transfusions to evaluate whether or not leukoreduction might improve outcomes among trauma patients, and found no difference in mortality or infectious morbidity among the 268 patients eligible for analysis[28]. Our clinical experience as described above demonstrates associations concerning both morbidity and mortality with older blood despite universal leukoreduction, further suggesting that the existence of a clinically relevant benefit of leukoreduction in the trauma setting remains doubtful.

## Summary

Although the growing body of literature demonstrating the deleterious effects of relatively old blood is compelling, we must be mindful that all of these reports have been retrospective, and most of these studies have evaluated patients who received a mixture of red cell units of varying storage age. As highlighted above, the difficulty in distinguishing the effect of storage age from the effect of transfusion volume in these studies is not insignificant. In our own work, we have employed statistical analysis that we feel best attenuates the potential residual confounding of transfusion volume. It remains quite possible, however, that prospective evaluation of the effect of storage age on outcome might yield contradictory results.

Certainly, prospective confirmation of the effect of blood storage on morbidity and mortality is now warranted. Schulman et al. attempted such a trial in the setting of a single-center Level 1 trauma center, randomizing patients to receive exclusively young (<11 days) versus old (>20 days) blood during the first 24 hours of hospitalization[29]. Unfortunately, in 1 year they were only able to enroll a small number of patients secondary to limitations of the blood bank. It is reasonable to expect that other institutions would face a similar challenge given the tight supply of blood. It is clear that only inter-institutional cooperation in the form of a multi-institutional trial will be successful in the recruitment of enough patients for a robust analysis. Hebert et al. performed a multi-center feasibility study in Canada, and reported that a large scale study would be feasible, but challenged by the maintenance of a sufficient blood supply to allow for randomization between old and young groups with a limited number of subsequent group crossovers[30]. Until such prospective studies have been completed and produce confirmative results, it would be premature to recommend any modification of current transfusion practice regarding storage age. Nonetheless, the implication that the transfusion of blood of relatively longer storage age may have negative consequences demands attention and, most importantly, further rigorous evaluation.

## Competing interests

The authors declare that they have no competing interests.

## Authors' contributions

MV and JW drafted the manuscript and performed critical revision. GM performed critical revision. All authors have read and approved the final manuscript.
